# Chelating agents for diluted geothermal brine reinjection

**DOI:** 10.1186/s40517-022-00227-1

**Published:** 2022-09-26

**Authors:** Jacquelin E. Cobos, Erik G. Søgaard

**Affiliations:** 1 Department of Mechanical and Electrical Engineering, University of Southern Denmark, Sønderborg, Denmark; 2 Department of Physics and Technology, University of Bergen, Bergen, Norway; 3 Department of Chemistry and Bioscience, Aalborg University, Copenhagen, Denmark

**Keywords:** SaltPower, Geothermal brine, Chelating agents, Isothermal titration calorimetry, Speciation simulations

## Abstract

“Blue energy” could be produced by exploiting the large salinity gradient between geothermal fluids and freshwater through a SaltPower system. This study is an attempt to select the most favorable chemicals to avoid injectivity issues when a diluted geothermal fluid resulting from the SaltPower system is returned to the reservoir. Three synthetic chelating agents (oxalic acid, EDTA, and EDDS) and one natural (humic acid) were evaluated through speciation simulations and isothermal titration calorimetry (ITC) experiments. The speciation simulation results indicate that the degree of complexing is highly dependent on pH and chelating agent type. The ITC experiments show that the total heat for the formation of soluble metal–ligand complexes in the rock + geothermal brine system follows: EDTA > EDDS > oxalic acid > humic acid. The simulations and calorimetry results suggest that EDTA could be used to avoid the precipitation of Fe(III) oxides and other minerals (e.g., calcite and dolomite) inside the porous media upon the reinjection of diluted geothermal brine coming from SaltPower electricity production.

## Introduction

The United Nations Framework Convention on Climate Change (UNFCCC) aims to “stabilize greenhouse gas concentrations in the atmosphere at a level that will prevent dangerous human interference with the climate system, in a time frame which allows ecosystems to adapt naturally and enables sustainable development” (Leggett 2020). The first international call to reduced carbon dioxide emissions ($\hbox {CO}_{2}$) and the presence of greenhouse gases (GHG) in the atmosphere was adopted in Kyoto, Japan 1997 and entered into force (international law) in 2005 (de Boer 2008). Once the Kyoto protocol was ended in 2012, the parties (industrialized nations) signed the Doha Amendment and subsequent the Paris Climate Agreement. The latter was adopted by nearly every nation (Leggett 2020).

The global awareness of climate change and its negative effects on global warming has increased a more sustainable energy production. However, in the twenty-first century, the worldwide energy mix is still dominated by fossil fuels even after an abrupt contraction in the Gross Domestic Product (GDP) and deep reductions in travel resulting from the Covid-19 pandemic. If the economic activity returns to pre-pandemic levels, the emissions could rebound as a consequence of the global energy demand. Under almost all sustainable development scenarios, renewable energies are projected to play a dominant role in new power generation by 2040 (Newell et al. 2020). Therefore, new alternative energy sources should be explored and embraced to mitigate the dangerous long-term effects of global climate change (Helfer et al. 2014).

SaltPower, osmotic power, or salinity gradient energy are relatively new energy conversion processes. Those terms refer to a sustainable energy production based on osmotic gradients resulting from mixing fluids with different salinity (Madsen et al. 2020; Cobos and Søgaard 2020, 2021). In the process of “blue energy” production, water molecules are transported spontaneously from the diluted fluid towards the concentrated saltier water due to osmotic forces. The resulting increased pressure can be easily used to produce energy by using a turbine (Skilhagen et al. 2008). The most well-known processes to harness this type of energy are pressure retarded osmosis (PRO) (Madsen et al. 2016; Yip and Elimelech 2012), reverse electrodialysis (RED) (Lacey 1980), and capacitive mixing (CapMix) (Yip et al. 2016). Different schemes have been investigated (e.g., mixing of seawater with river water, desalination brine with treated wastewater, concentrated salt brine with brackish water), but it has not yet reached a commercial level (Madsen et al. 2020).

The most promising scheme for salinity gradient energy production is the usage of concentrated brines (hypersaline sources) that results in high energy densities (Sidney 2001). Madsen et al. (2017) demonstrate that the PRO process can be operated at pressures up to 70 bar with power densities above 5 W/$\hbox {m}^{2}$. In a later publication, Madsen et al. (2020) showed that osmotic power production can be combined with geothermal heat mining. The synergy between both systems can lead to several advantages, including reduction of the brine viscosity, coverage of the electricity that drives internal processes at geothermal plants (e.g., heat, production, and reinjection pumps). Moreover, both technologies can share capital costs for their development, maintenance, labor, and monitoring.

The idea of combining heat mining with salinity gradient energy production is feasible. However, as shown in our previous works the oxidation and precipitation of iron as Fe(III) oxides compromise the overall reservoir assurance (Cobos and Søgaard 2020, 2021). As concluded in Cobos and Søgaard (2020), the reinjection of diluted brine from a PRO process is a viable option only if iron is kept under control. This is because half-diluted geothermal brine could contain oxygen which oxidizes Fe(II). Citric acid was tested and proved as a potential alternative for geothermal brine reinjection. This agent keeps iron in solution due to its chelating characteristics and also improves the rock properties (porosity and permeability) as reported by Cobos and Søgaard (2021). Those authors obtained a porosity increment of more than 24% after half-diluted brine with citric acid was injected into Berea sandstone core plugs. The brine permeability was also improved even after a pause in the fluid injection. According to Cobos and Søgaard (2021), the rock properties were improved because of the dissolution of carbonate cements (siderite and ankerite) present in Berea sandstone.

**Fig. 1:**
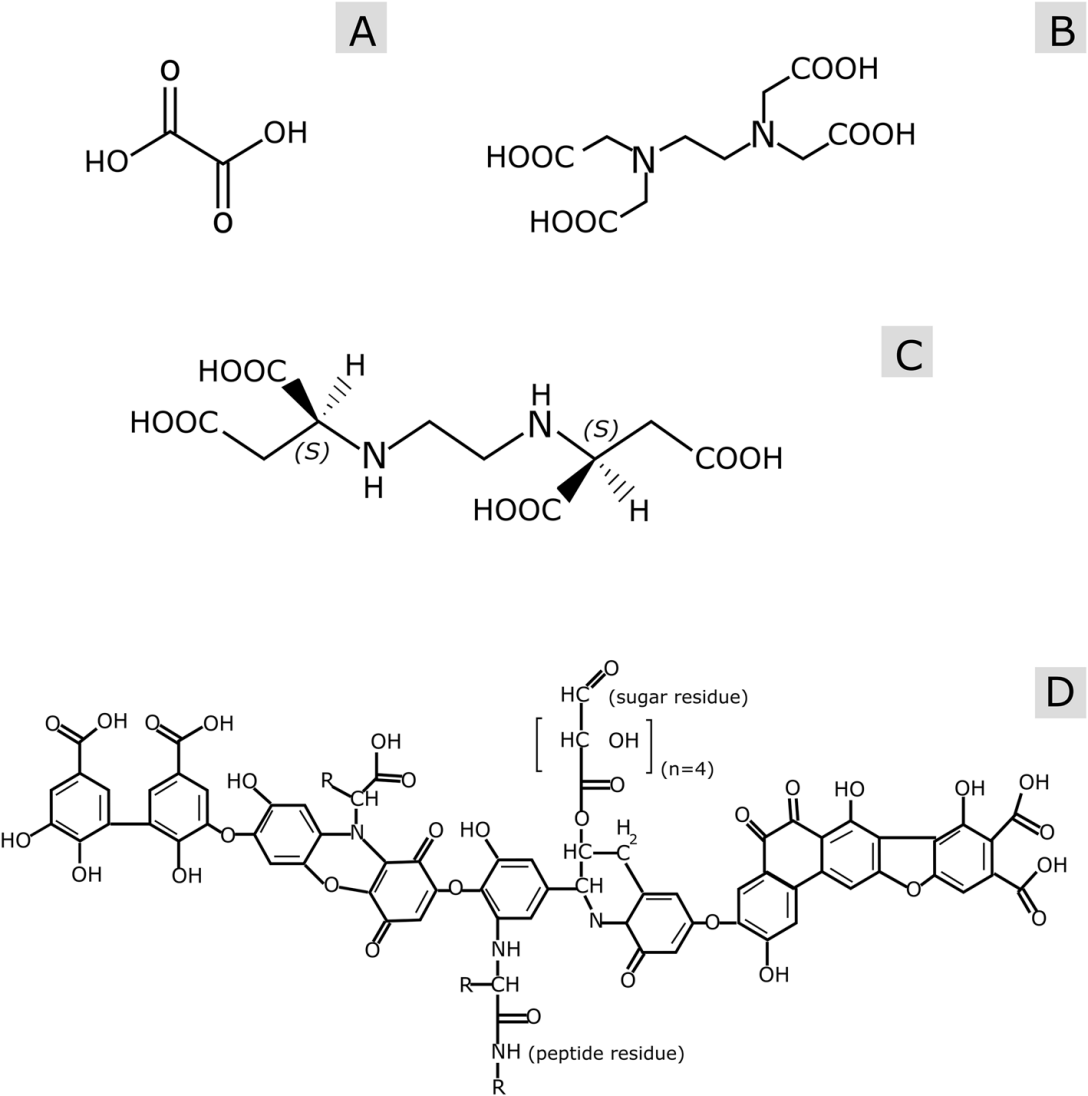
Structures of chelating agents used in the present study. Chemical structure of **a** oxalic acid, **b** EDTA, **c** [S,S]-EDDS, and **d** humic acid

The present research aimed to determine the feasibility of using different chelating agents to avoid injectivity issues when the fluid resulting from the combined geothermal heat and pressure retarded osmosis energy production (SaltPower electricity generation) is returned to the reservoir. Note that injectivity is defined as the rate of fluid injection over the differential pressure between producer and injector in barrels per day per pounds per square inch (bbl/d/psi) (Reservoir Engineering 2016). The agents used in this study are polycarboxylate (oxalic acid), aminopolycarboxylic acids (ethylenediaminetetraacetic acid—EDTA, ethylenediamine-S,S′-disuccinic acid—EDDS) and humic substances (humic acid). The chemical structure of those chelates is presented in Fig. 1.

As observed, those agents possess at least two functional groups capable of combining with the metals present in the diluted geothermal brine by donating a pair of electrons. This in turn, allows the formation of stable heterocyclic ring structures in which the metal ion is gripped firmly. Speciation simulations and isothermal calorimetry experiments (ITC) were used to determine the feasibility of the reinjection of the diluted geothermal brine with chelating agents. The present work not only confirms, but explains from a calorimetric point of view, that the usage of chelating agents is a viable solution to avoid the precipitation of ferric hydroxide or other iron-containing compounds that may clog the pores in the reservoir and lead to injectivity problems. Moreover, iron could also oxidize and precipitate in the surface installations of geothermal power plants. To the best of our knowledge, no existing literature has investigated the rock–fluid and fluid–fluid interactions that takes place during the reinjection of a diluted geothermal brine with chelating agents from a microcalorimetric point of view. This work fills this gap and provides relevant insights of the complex interactions that could take place if a diluted geothermal brine with chelating agents is reinjected into a sandstone reservoir.

## Materials and methods

### Rock material

Similar to previous works (Cobos and Søgaard 2020, 2021), Berea sandstone was used for the isothermal titration calorimetry (ITC) experiments. This is because Berea sandstone is a homogeneous reference rock, whose grains are well-sorted and composed of quartz held together by silica (Churcher et al. 1991).

The elemental composition of Berea sandstone was determined by a Rigaku supermini 200-XRF equipment and the mineralogical composition through a PANalytical X-ray Powder Diffraction instrument (XRD). Particles with a size of < 100 μm from a crushed Berea sandstone core plug were used for the XRF and XRD analysis.

### Brines

A highly saline brine from Thisted geothermal energy plant in Denmark was used for the experiments presented in this study. Thisted geothermal plant was the first in the country and the only one out of three (Thisted, Margretheholm, Sønderborg) that has run continuously since it was commissioned in 1984 by Dansk fjernvarme. The geothermal plant can produce up to 7 MW of heat from 15% saline water extracted from Gassum sandstone aquifer at 1.23 km depth, which is combined with 10 MW of driving heat coming from a waste incineration plant and a straw fired boiler. The plant has a production and injection wells, which are separated by a vertical distance of 1.5 km. In the production well, a submergible pump located at 400 m of depth pumps up to 200 $\hbox {m}^{3}$/h to the surface. Thereafter, the fluid passes through sand filters to remove harmful particles before being send trough two absorption heat pumps that cool the fluid down to 12 °C and transfer the heat to the local district heating network. After heat extraction, the geothermal fluid is pumped back through an injection well to gradually refill the large hot water reservoir (Cobos and Søgaard 2020; Mahler et al. 2013). Table 1 shows the ionic composition and intrinsic properties of Thisted geothermal brine (TB) and half-diluted Thisted geothermal brine (2D * TB). The ionic composition of the geothermal brine was determined through inductively coupled plasma-optical emission spectrometry (ICP-OES) and ion chromatography (IC). On the other hand, the intrinsic properties (electrical conductivity, salinity, pH, and density) were determined using a PC 2700 meter from Eutech instruments and a DMA 35 Anton Paar density meter (Cobos and Søgaard 2020).

**Table 1:** Ionic composition in mmol/l, ionic strength $I_\text{c}$ in mol/l, electrical conductivity (EC) in mS/m, total dissolved solids (TDS) in ppm, density ($\rho$) in g/cm^3^, and pH

	Ionic composition									Intrinsic properties				
	$\text{K}^+$	$\text{Na}^+$	$\text{Sr}^{2+}$	$\text{Mg}^{2+}$	$\text{Ca}^{2+}$	$\text{SO}_4^{2-}$	$\text{Cl}^-$	$\text{HCO}_3^-$	Fe(*II*)	$I_\text{c}$	EC	TDS	$\rho$	pH
TB	17.8	1999	3.6	64.0	143	1.2	2728	0.5	1.6	2.8	190	176000	1.11	6.71
2D * TB	10.4	1245	1.9	33.1	88	0.6	1621	0.5	0.9	1.7	110	103000	1.05	6.69

As stated in previous works (Cobos and Søgaard 2020, 2021), the main issue with the reinjection of diluted geothermal brine coming from SaltPower electricity generation was the oxidation and precipitation of iron oxides inside the porous medium. A plausible option to avoid this problem is the addition of chelating agents in the diluted geothermal brine coming from the SaltPower unit to keep iron in solution. In the present work, four different multidentate complex formers were evaluated; three synthetic organic chelating agents (oxalic acid, EDTA, and EDDS) and one natural chelate (humic acid). Oxalic acid is a bidentate agent (Lewis bases that donates two electron pairs, bi, of electrons to a metal atom) with two coordination positions that binds simultaneously to the metal ion, forming a single chelate ring (Zhang and Zhou 2019). EDTA and EDDS are hexadentate agents with six coordination positions made of four oxygen donor atoms from the carboxyl groups and two nitrogen donor atom (Stumm 1992). Those agents are capable of complexing with metal atoms to form five interlocking chelate rings (Prete et al. 2021). The last chelating agent evaluated in this work was humic acid, a complex substance that resulted from the conversion of lignin from plant materials. It contains hydroxyl-, phenoxyl-, and carboxyl- reactive groups (Boguta and Sokolowska 2013).

The chelating ability of a ligand to keep Fe(II) in solution was quantified by adding separately 1.5 moles of oxalic acid ($\hbox {C}_2\hbox {H}_2\hbox {O}_4$), EDTA ($\hbox {C}_1\text{OH}_14\hbox {N}_2\hbox {Na}_2\hbox {O}_8$ $\cdot$ 2$\hbox {H}_2\hbox {O}$), EDDS ($\hbox {C}_1\hbox {OH}_16\hbox {N}_2\hbox {O}_{8}$), and humic acid per measured mole of Fe(II) to the half-diluted geothermal brine, which are represented by 2D * $\hbox {TB}_{\mathrm{oxalic}}$, 2D * $\hbox {TB}_{\mathrm{EDTA}}$, 2D * $\hbox {TB}_{\mathrm{EDDS}}$, and 2D * $\hbox {TB}_{\mathrm{HAs}}$, respectively. In other words, 2.4 mmol/l of oxalic acid, EDTA, EDDS, and humic acid were added to TB since the brine has an iron concentration equal to 1.6 mmol/l. The same concentration of the chelating agents was added to deionized water (DI), which are named as $\hbox {DI}_{\mathrm{oxalic}}$, $\hbox {DI}_{\mathrm{EDTA}}$, $\hbox {DI}_{\mathrm{EDDS}}$, and $\hbox {DI}_{\mathrm{HAs}}$. Table 2 displays pH values for the fluids used in the microcalorimetric experiments.

**Table 2:** pH values for brines with chelating agents (2D * $\hbox {TB}_{\mathrm{chelating}}$) and deionized water with chelating ($\hbox {DI}_{\mathrm{chelating}}$)

	2D * $\hbox {TB}_{\mathrm{oxalic}}$	$\hbox {DI}_{\mathrm{oxalic}}$	2D * $\hbox {TB}_{\mathrm{EDTA}}$	$\hbox {DI}_{\mathrm{EDTA}}$	2D * $\hbox {TB}_{\mathrm{EDDS}}$	$\hbox {DI}_{\mathrm{EDDS}}$	2D * $\hbox {TB}_{\mathrm{HAs}}$	$\hbox {DI}_{\mathrm{HAs}}$
pH	2.59 ± 0.21	2.68 ± 0.01	2.59 ± 0.21	2.96 ± 0.58	6.18 ± 0.02	9.44 ± 0.20	6.25 ± 0.25	7.53 ± 0.02

### Speciation simulations

The degree of complexation of the chelating agents with the metal ions in 2D * TB was determine through Visual MINTEQ, public-domain software for the calculation of metal speciation, solubility equilibria, sorption for natural waters (Gustafsson 2022). Briefly, the speciation modeling simulations consisted in defining pH, ionic strength, and 2D * TB temperature. Then, the components of the geothermal brine and the chelating agents (oxalate, EDTA, humic acids), as well as their concentrations were specified. Note that humic acids were added as organic material using the Stockholm Humic Model (SHM), which provides a realistic assessment of metal–humic complexes (Gustafsson 2001). Due to the high salinity of the geothermal brine, Brønsted–Guggenheim–Scatchard specific ion interaction theory (SIT) was used to determine the activity coefficients. The SIT model, a simplified version of Pitzer, can be used for ionic strengths up to 4 M (Bretti et al. 2004). As observed in Table 1, the ionic strength ($I_\mathrm{c}$) of half-diluted Thisted brine is lower than 2 M, and therefore the SIT model is suitable for the simulations.

“Spana” software, which is equivalent to Medusa/Hydra was used to simulate the distribution of iron species as function of pH. This software was developed by the Royal Institute of Technology in Sweden to draw chemical-equilibrium diagrams based on the HaltaFall algorithm (Puigdomènech et al. 2014). Notice that the equilibrium constants for EDDS and humic acids were not available in the Spana Database, hence those chelating agents were not included in the equilibrium diagrams. For the simulations, the concentration of the chelating agents (oxalic acid and EDTA) was 2.42 mM. This is because 1.5 moles of those agents should be added to the geothermal brine to keep Fe(II) in solution (Cobos and Søgaard 2021). The Spana simulations also took into consideration other parameters, such as Fe(II) concentration equal to 0.9 mM, ionic strength of 1.7 mM $\simeq$ 0.002 M, and ambient temperature (25 °C).

### Microcalorimetry experiments

A multichannel microcalorimetric system Tam IV from TA Instruments was used to determine the interactions between the Berea sandstone powder and Thisted geothermal brine (TB) with different chelating agents. The TAM IV apparatus has a precision of ± 100 nW, and a thermostat with an accuracy of ± 0.1 °C. The microcalorimetry experiments consist of placing 100 mg of Berea sandstone particles in a stainless steel cell (reaction vessel) and adding 200 μl of TB to those particles to resemble a geothermal reservoir. Thereafter, the ampule with the slurry of the sandstone particles and TB is lowered stepwise into the microcalorimeter until it reaches its final position. After 1 h of equilibrium time at 50 °C, 10 injections of 9.948 μl of the selected brine (2D * $\hbox {TB}_{\mathrm{oxalic}}$, 2D * $\hbox {TB}_{\mathrm{EDTA}}$, 2D * $\hbox {TB}_{\mathrm{EDDS}}$, 2D * $\hbox {TB}_{\mathrm{HAs}}$) are titrated into the sandstone particles wetted with TB. The time interval between each injection is 600 s. Fluid–fluid interactions were performed in a similar manner but without the sandstone particles in the reaction vessel. In other words, 2D * $\hbox {TB}_{\mathrm{chelating}}$ was added directly into TB. Blank experiments were also carried out in order to assess the degree of in situ geothermal brine dilution. Those experiments consisted in adding $\hbox {DI}_{\mathrm{chelating}}$ into either Berea + TB or TB. A summary of the microcalorimetric experiments performed in this study is presented in Table 3. As observed, EDTA decrease pH of the half-diluted brine which can decrease the oxidation rate of Fe(II) and increase the solubility of ferrihydrite.

**Table 3:** Overview of microcalorimetry evaluation

Experiments		Oxalic	EDTA	EDDS	HAs
Rock–fluid interaction					
2D * $\hbox {TB}_{\mathrm{chelating}}$ into Berea + TB		X	X	X	X
$\hbox {DI}_{\mathrm{chelating}}$ into Berea + TB (blank)		X	X	X	X
Fluid–fluid interaction					
2D * $\hbox {TB}_{\mathrm{chelating}}$ into TB		X	X	X	X
$\hbox {DI}_{\mathrm{chelating}}$ into TB (blank)		X	X	X	X

NanoAnalyze™ software from TA Instruments was used to analyze the results from the ITC experiments.

## Results and discussion

### Chemical and mineralogical composition of the rock material

The elemental composition results of Berea sandstone obtained by X-ray fluorescence (XRF) are shown in Table 4, where concentration is displayed in the form of metal oxides. The rock material is predominantly composed of silicon (Si), aluminum (Al), and potassium (K). Other metals, such as iron (Fe) and magnesium (Mg) are also present, which could indicate that the rock material is composed of small tracers or impurities (e.g., ankerite and siderite).

**Table 4:** The results of X-ray fluorescence (XRF) analysis of Berea sandstone

Compound	$\hbox {SiO}_{2}$	$\hbox {Al}_2\mathrm{O}_{3}$	$\hbox {K}_{2\mathrm{O}}$	$\hbox {Fe}_2\mathrm{O}_{3}$	$\hbox {MgO}$	$\hbox {TiO}_{2}$	$\hbox {CaO}$	Cl
Mass (%)	81.40	13.80	2.00	0.98	0.70	0.55	0.32	0.11

**Fig. 2:**
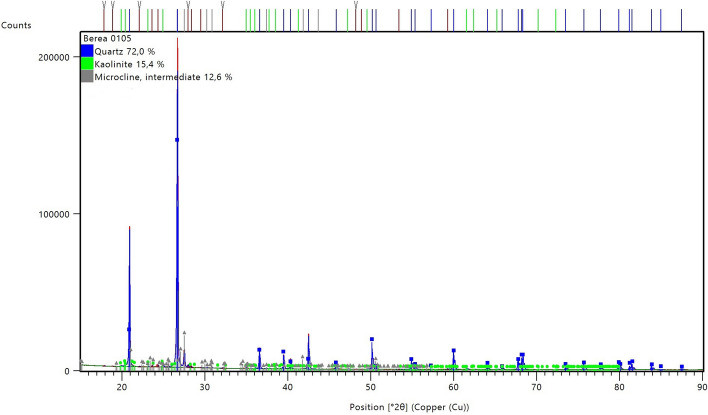
The results of X-ray powder diffraction (XRD) analysis of Berea sandstone material. Blue line: quartz, red: kaolinite, and green: k-feldspar

The X-ray powder diffraction (XRD) pattern for Berea sandstone is shown in Fig. 2. As observed, the identified minerals are quartz, kaolinite and k-feldspar. However, tracer minerals have not been identified due to the small size of the peaks in the XRD pattern. Based on the XRF results, the rock material can also contain ankerite, siderite, mica, illite, chlorite and calcite, which is in line with different studies reported in the literature (Dawson et al. 2015; Cerasi et al. 2016).

### Speciation simulations of chelating agents in two times diluted geothermal brine

**Fig. 3:**
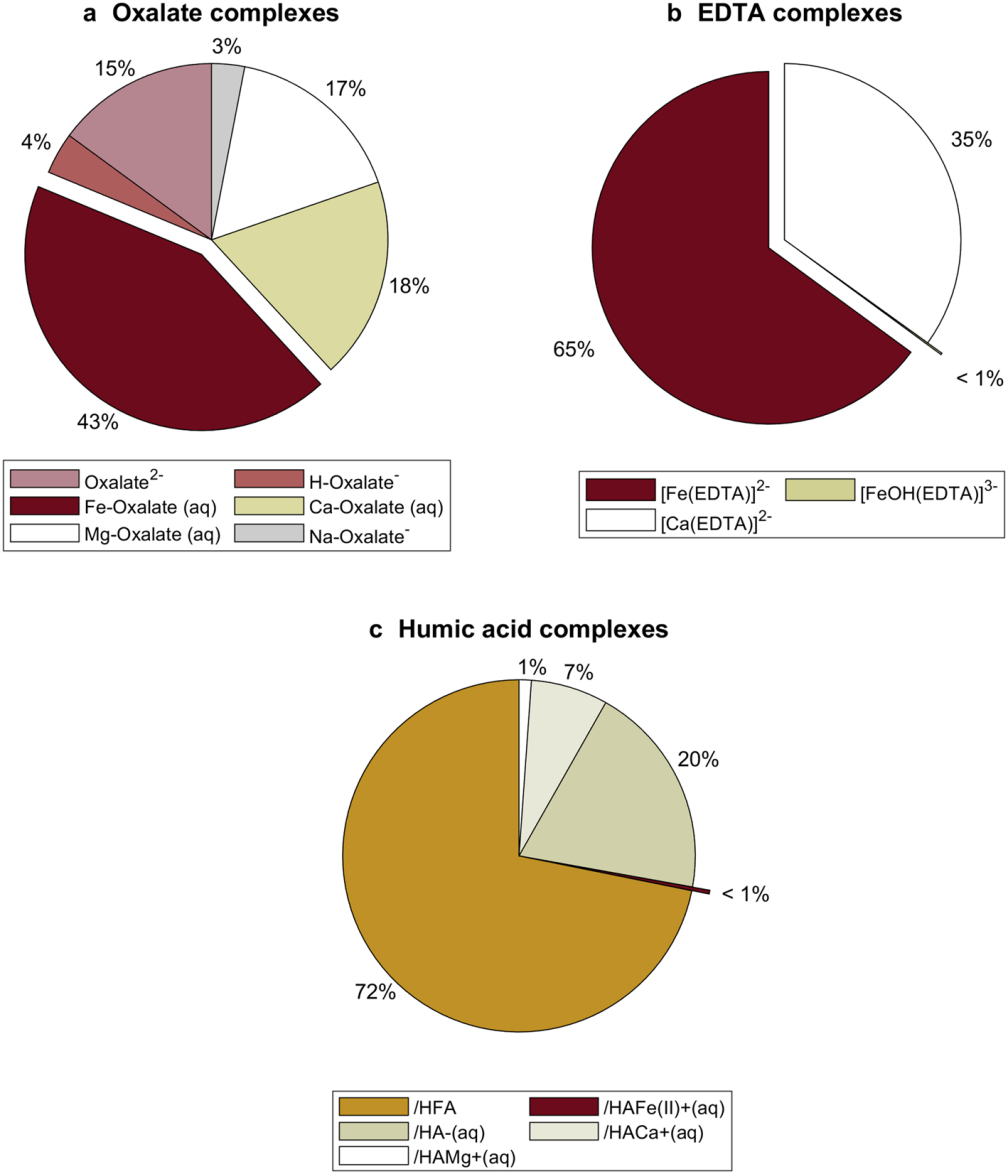
Species distribution for chelating agents at 25 °C

The species distribution when oxalic acid, EDTA, and humic acids were added separately to 2D * TB is presented in Fig. 3. The results from the simulations shows that the synthetic chelating agents (oxalic acid, and EDTA) complexed to a great extent with iron. In this sense, 43% was complexed with oxalic acid forming Fe-oxalate (aq), 65% with EDTA forming $\hbox {FeEDTA}^{{2-}}$. On the contrary, the iron complexation by humic acids is less than 1%. A lesser amount of alkali and alkaline earth cations (Na, Ca, Mg and K) was bound to humic acids because those cations are held as counter ions to the organic matter (Tipping 2002). It is important to highlight that 15% of oxalic acid and 75% of humic acid remained uncomplexed (free ligand) in 2D * $\hbox {TB}_{\mathrm{oxalic}}$ and 2D * $\hbox {TB}_{\mathrm{humic}}$, respectively. Consequently, those free ligands could interact with the in situ geothermal brine.

The speciation simulations showed that the tested chelating agents (oxalic acid, EDTA, and humic acids) complexed with the metals in the diluted geothermal brine. The distribution of different metals with respect of free ions and aqueous metal-complexes is given by the equilibrium constants. As reported in the supplementary manual of Visual MINTEQ (HydroGeoLogic and Allison Geoscience 1999), the stability constant for EDTA is the highest among the chelating agents. In other words, the EDTA metal complexes are significantly more stable than the other chelating-metal complexes.

**Fig. 4:**
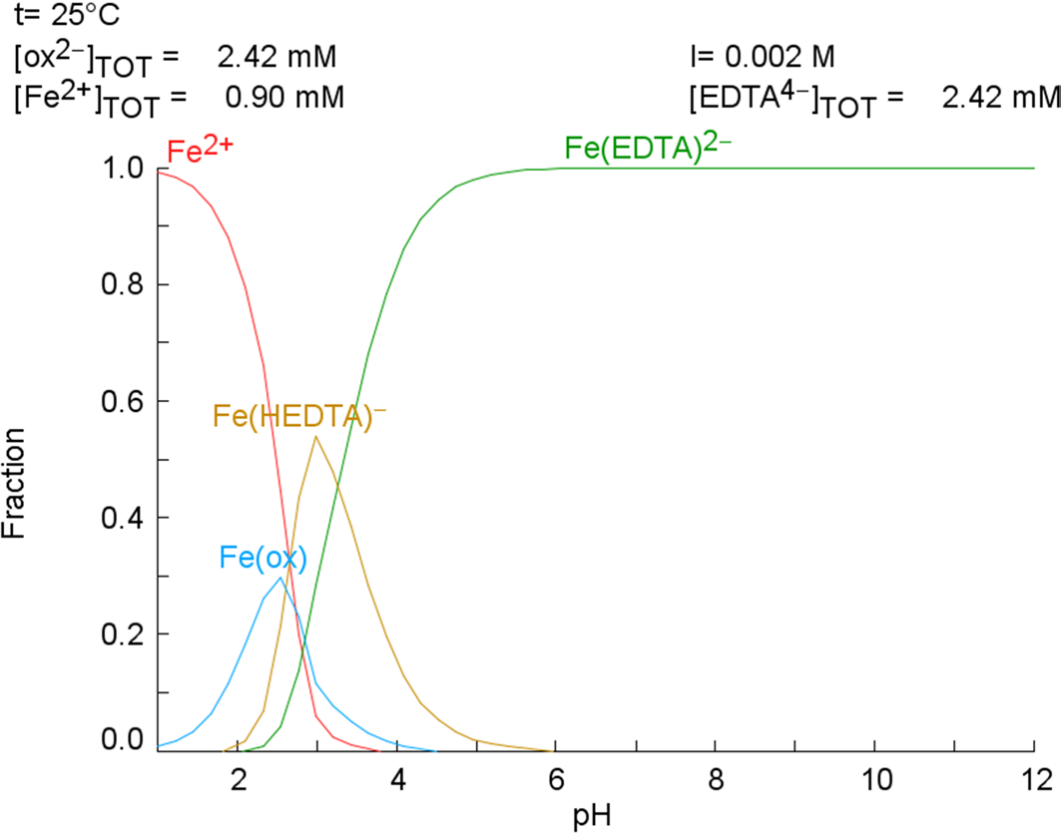
Distribution diagram of iron species as a function of pH. Drawn using Spana software, Royal Institute of Technology, Sweden

Figure 4 presents the distribution of iron species as function of pH (values ranging from 1 to 12) obtained by “Spana”. As observed, Tris(oxalato)ferrate(III) ion, [Fe(ox)], could be formed with the oxalate bidentate ligand in a pH range between 1 and 4. The maximum fraction of Fe(II) that could be complexed by oxalic acid is 0.3, which is in line with the results presented in Fig. 3. In the case of the hexadentate EDTA ligand two complexes, [Fe$\hbox {(HEDTA)}^{-}$] and [Fe$\hbox {(EDTA)}^{{2-}}$], could be obtained in a wider pH range. The formation of [Fe$\hbox {(EDTA)}^{{2-}}$] increases with pH, being maximum at a value of 5. Thereafter, the concentration of that complex is stable. [Fe$\hbox {(HEDTA)}^{-}$] complex, on the other hand, requires low pH values; maximum at a pH 3.5 and then its concentration decreases. The chemical equilibrium diagram clearly shows that EDTA is more prone to form complexes with iron than oxalic acid.

### Addition of diluted geothermal brine with chelating agents into Berea sandstone–Thisted brine system

Isothermal titration calorimetry was used to get physico-chemical insights into the processes that could take place if a diluted geothermal brine with different chelating agents (oxalic acid, EDTA, EDDS, and humic acid) is reinjected into a geothermal reservoir. Note that the main processes that could take place due to the injection of diluted geothermal brine with different chelating agents are mineral dissolution, disruption of the hydrogen bonding due to dilution of the in situ brine, and reaction with metal ions to form soluble chelate complexes. The chelating agents added to two times diluted Thisted brine (2D * $\hbox {TB}_{\mathrm{oxalic}}$, 2D * $\hbox {TB}_{\mathrm{EDTA}}$, 2D * $\hbox {TB}_{\mathrm{EDDS}}$, 2D * $\hbox {TB}_{\mathrm{HAs}}$) were injected separately into Berea sandstone particles wetted with the original formation brine (TB).

**Fig. 5:**
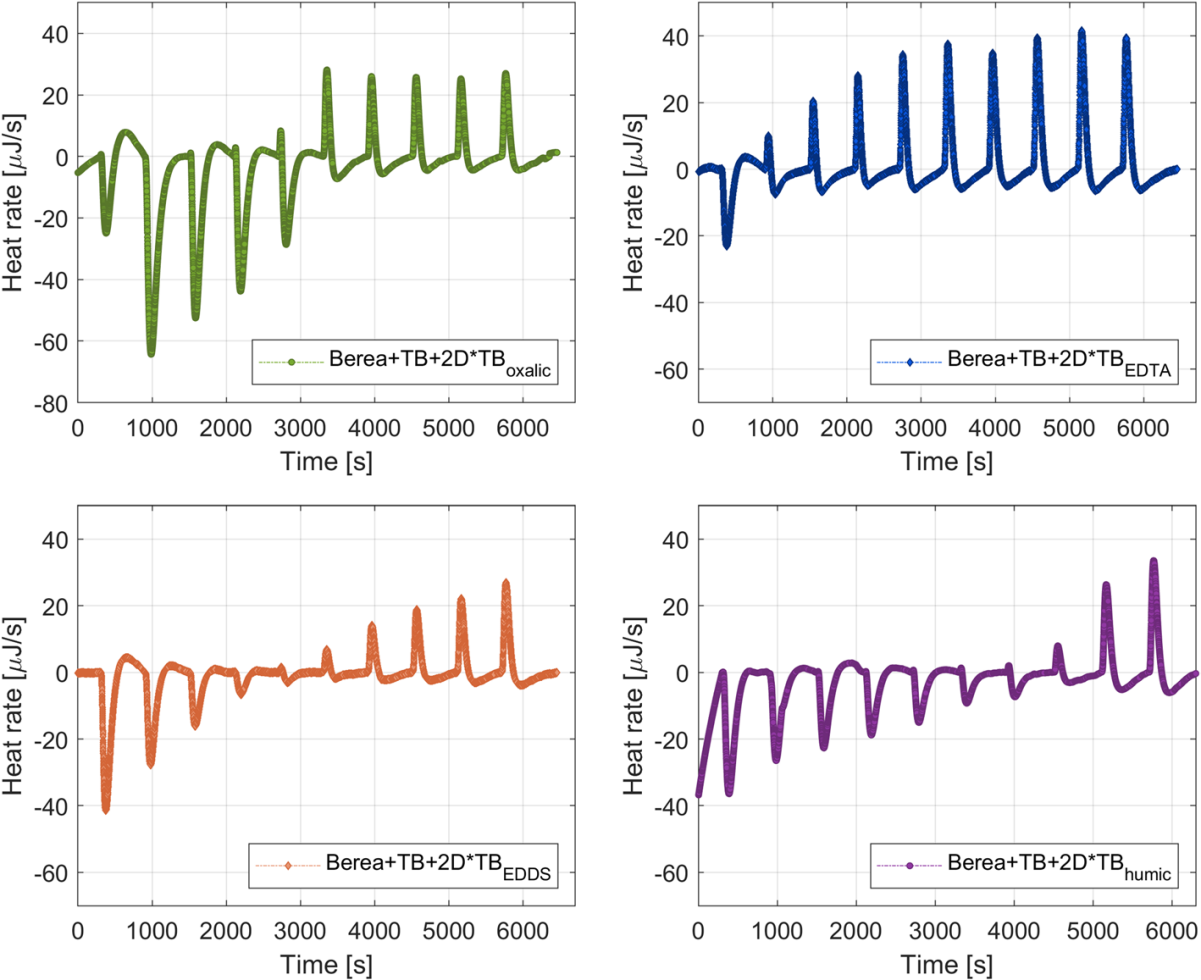
Thermograms for the interaction of 2D * $\hbox {TB}_{\mathrm{chelating}}$ and Berea + TB system

The heat flow signal recorded by the TAM IV apparatus when the chelating agents in 2D * TB were injected into the Berea sandstone particles wetted with TB (formation brine) is presented in Fig. 5. As observed, the interaction of 2D * $\hbox {TB}_{\mathrm{chelating}}$ with the Berea+TB system is partially endothermic (peaks down) and partially exothermic (peaks up).

The endothermic peaks observed in Fig. 5 could be associated with a disruption of the second and third hydration shells around the single ionic species caused by dilution (Mancinelli et al. 2007) and the exothermic peaks might be related to the formation of soluble chelate complexes. The endothermic peaks could also be related to the interaction between the tested chelating agents (oxalic, EDTA, EDDS and humic acid) and the rock particles. The dissolution of carbonate bearing cement (part of the Berea sandstone particles) might have contributed to the endothermic response as it was observed previously in Cobos and Søgaard (2021). Frenier et al. (2000), Moghadasi et al. (2007) mentioned that EDTA-type chelating agents can be used as dissolution agents in matrix acidizing of carbonate formations and also as scale removers due to the combined influence of chelation and hydrogen ion attack. The possibility of using diluted geothermal brine with chelating agents could be a good alternative to overcome the very often met scaling problems in the reinjection wells. As shown in a previous work (Cobos et al. 2021), an excess of $\hbox {Ca}^{{2+}}$ and $\hbox {SO}_4^{{2-}}$ could lead to the formation and precipitation of carbonate and sulfate scales. This in turn causes a significant permeability reduction in the reservoir, which can lead to the shutdown of the reinjection well. Consequently, additional wells and/or sidetracks should be targeted due to the declining transmissivity.

**Fig. 6:**
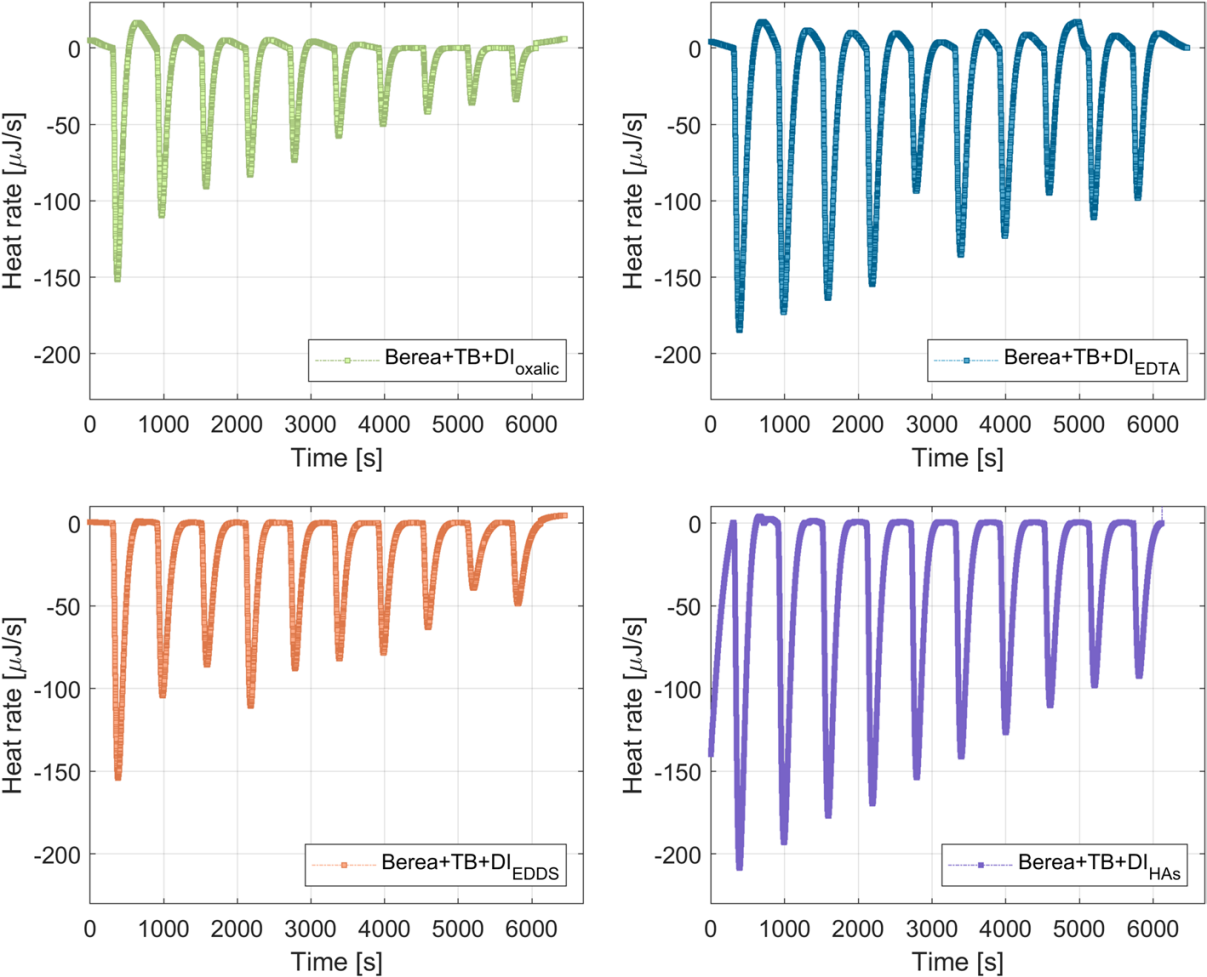
Thermograms for the interaction of $\hbox {DI}_{\mathrm{chelating}}$ and Berea + TB system

In order to test the extreme dilution of the geothermal brine that is in contact with the rock particles, deionized water with chelating agents ($\hbox {DI}_{\mathrm{oxalic}}$, $\hbox {DI}_{\mathrm{EDTA}}$, $\hbox {DI}_{\mathrm{EDDS}}$, and $\hbox {DI}_{\mathrm{humic}}$) was added separately into the Berea + TB system. Figure 6 displays the heat response registered in the microcalorimeter apparatus for the injection of DI with chelating agents into the Berea + TB system. It is noted that the dilution of the geothermal brine masked the chelation process, in which the central metal ion is surrounded by a ring-type structure that resembles a claw (Moghadasi et al. 2007).

The heat developed by each injection of 9.947 μl of 2D * $\hbox {TB}_{\mathrm{chelating}}$ or $\hbox {DI}_{\mathrm{chelating}}$ into the Berea + TB system was obtained by the integration of the heat rate observed in Figs. 5 and 6 over the baseline (reference time to compare all the titrations). Table 5 summarizes the results from the integration of the area between peaks and the baseline. It is observed in Table 5 that 2D * $\hbox {TB}_{\mathrm{EDTA}}$ attains the highest exothermic response. It was found a total exothermic energy for 2D * $\hbox {TB}_{\mathrm{oxalic}}$ of − 2.47 mJ, 2D * $\hbox {TB}_{\mathrm{EDTA}}$ − 8.36 mJ, 2D * $\hbox {TB}_{\mathrm{EDDS}}$ − 3.24, and 2D * $\hbox {TB}_{\mathrm{humic}}$ − 1.96, respectively. Therefore, the interaction of 2D * $\hbox {TB}_{\mathrm{chelating}}$ with Berea + TB follows: EDTA > EDDS > Oxalic > humic acid.

**Table 5:** Heat values for 2D * $\hbox {TB}_{\mathrm{chelating}}$ or $\hbox {DI}_{\mathrm{chelating}}$ into Berea + TB (rock–fluid interactions)

injection	Oxalic system (mJ)		EDTA system (mJ)		EDDS system (mJ)		Humic system (mJ)	
	2D * $\hbox {TB}_{\mathrm{oxalic}}$	$\hbox {DI}_{\mathrm{oxalic}}$	2D * $\hbox {TB}_{\mathrm{EDTA}}$	$\hbox {DI}_{\mathrm{EDTA}}$	2D * $\hbox {TB}_{\mathrm{EDDS}}$	$\hbox {DI}_{\mathrm{EDDS}}$	2D * $\hbox {TB}_{\mathrm{Humic}}$	$\hbox {DI}_{\mathrm{Humic}}$
1	0.62	12.24	1.57	21.29	3.53	20.11	4.65	28.04
2	9.86	10.57	0.93	21.98	2.76	14.42	3.16	27.67
3	5.27	8.74	0.41	21.08	1.80	12.02	2.10	25.03
4	4.76	7.32	− 0.60	19.69	0.72	13.67	1.99	23.52
5	2.97	6.46	− 0.69	12.29	0.35	11.23	1.71	21.06
6	0.01	5.42	− 1.21	15.86	− 0.07	10.36	1.26	19.63
7	− 0.32	5.15	− 1.38	13.93	− 0.47	9.92	1.07	17.63
8	− 0.74	4.44	− 1.64	7.63	− 0.80	8.49	0.42	15.49
9	− 0.68	3.83	− 1.65	12.24	− 0.94	6.26	− 0.76	14.01
10	− 0.74	3.53	− 1.19	8.95	− 0.96	7.63	− 1.19	13.62

The enthalpy change per unit ionic strength ($\Delta$*H*) can be estimated by Eq. 1 (Cobos et al. 2018; Cobos 2020) since those rock + geothermal brine systems are at isobaric and isothermal conditions. In that equation, [$Q_{\mathrm{inj}}$] is the heat developed by each injection, [IS] is the ionic strength of the mixture in mmol/l and $V_{\mathrm{i}}$ is the volume of each titration:

1 $$\begin{aligned} Q_{\text{inj}} = \Big (\Delta H \times [\text{IS}] \times V_{\text{i}}\Big ). \end{aligned}$$

**Fig. 7:**
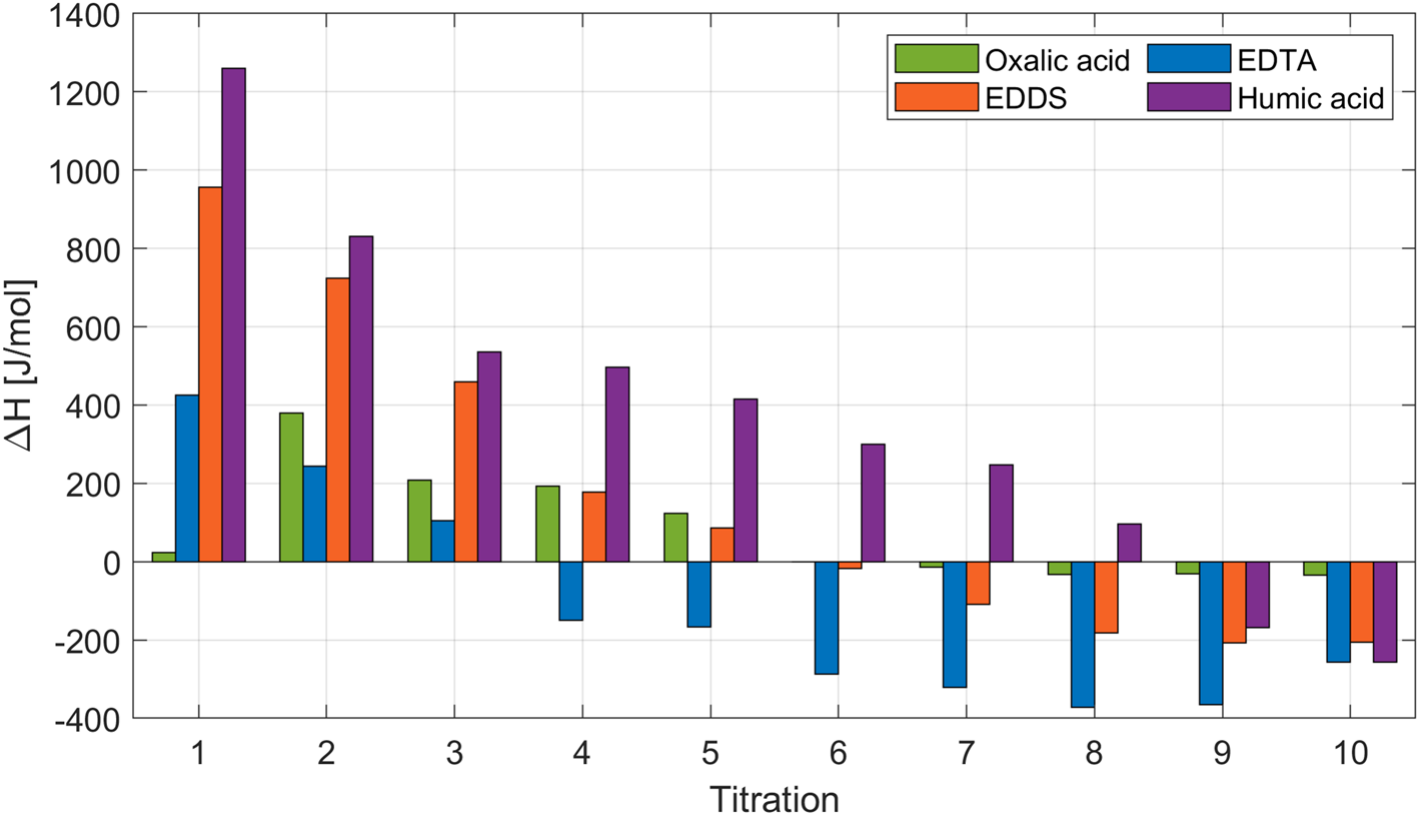
Enthalpy change ($\Delta$*H*) values for 2D * $\hbox {TB}_{\mathrm{chelating}}$ into Berea + TB

The enthalpy values ($\Delta$*H*) for the interaction of the 2D * $\hbox {TB}_{\mathrm{chelating}}$ with the sandstone particles impregnated with formation brine (TB) are presented in Fig. 7.

As observed in Fig. 7, EDTA in 2D * TB gives more negative enthalpy values (highest exothermic response) than the other chelating agents. This is in accordance with the general scientific consensus (Prete et al. 2021; Zhang and Zhou 2019; Weber 1996; Boggs et al. 1985). EDTA is a strong chelating agent able to complex with metals in 1:1 when fully deprotonated, the ligand occupies the corners of an octahedron and the metal atom is located in the center. The strong chelating ability of EDTA could be associated with its coordination number, which is six due to its functional groups (four carboxylic acid and two amine) (Stumm and Morgan 1996). The addition of 2D * $\hbox {TB}_{\mathrm{EDDS}}$ into the Berea + TB system shows the second highest exothermic response but smaller in comparison with EDTA. This could be because EDDS has similar metal chelating properties than EDTA since it is a structural isomer with two chiral centers and asymmetry plane (Zhang and Zhou 2019; Prete et al. 2021). Oxalic acid, bidentate chelator (coordination number equal to two), gives a lower exothermic enthalpy than EDTA and EDDS since it only forms a single chelate ring. The lowest exothermic but highest endothermic enthalpy was obtained for 2D * $\hbox {TB}_{\mathrm{humic}}$. The large endothermic response could be associated with changes in conformational properties of the humic substances upon binding metal ions. The humic substances have different active sites, non-specific weak sites formed by oxygen-containing ligands (abundant), stronger sites formed by combinations of oxygen, nitrogen, and sulphur (less abundant). At those sites, cations can be bound and therefore the intramolecular charge repulsion will be reduced. This in turn disrupts the macro-molecule that collapses and expels water (Tipping 2002).

### Addition of diluted geothermal brine with chelating agents into Thisted brine

**Fig. 8:**
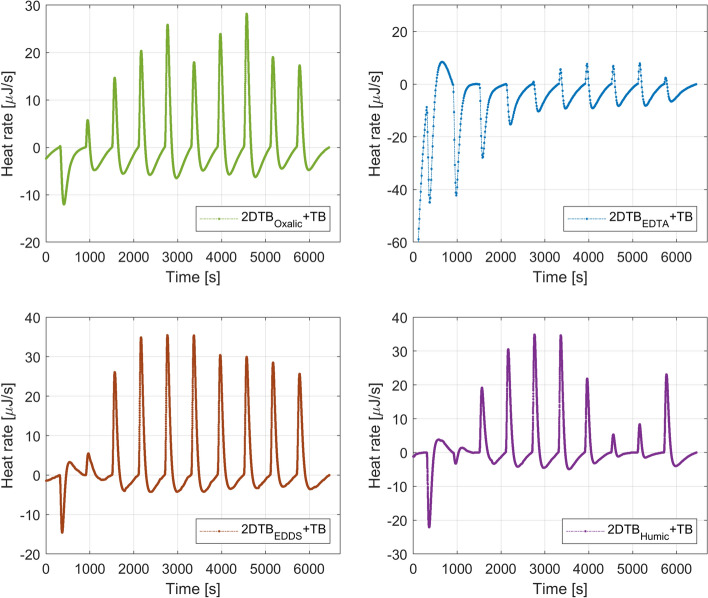
Thermograms for the interaction of 2D * $\hbox {TB}_{\mathrm{chelating}}$ and TB

Physicochemical interactions that occur at the fluid–fluid interface were also studied through microcalorimetry. Figure 8 shows the heat flow signal for the injection of diluted geothermal brine with chelating agents (2D * $\hbox {TB}_{\mathrm{oxalic}}$, 2D * $\hbox {TB}_{\mathrm{EDTA}}$, 2D * $\hbox {TB}_{\mathrm{EDDS}}$, 2D * $\hbox {TB}_{\mathrm{HAs}}$) into TB.

As observed in Fig. 8, the interaction between 2D * $\hbox {TB}_{\mathrm{oxalic}}$ and TB is both partially endothermic and exotermic. This is in accordance with the previous experiments in which 2D * $\hbox {TB}_{\mathrm{chelating}}$ was injected into Berea + TB. As observed, the sizes of the peaks in the thermograms displayed in Fig. 8 are much smaller than the ones presented in Fig. 5. This confirms that the tested chelating agents caused the dissolution of the iron bearing-carbonate cement that is also part of the Berea sandstone particles. Rosenbrand et al. (2015) indicated that Berea sandstone contains ankerite [$\hbox {Ca(Fe, Mg)} (\hbox {CO}_3)_2)$] and siderite ($\hbox {FeCO}_{3}$). Those minerals could have been dissolved by a combined chelating effect and $\hbox {H}^{+}$ attack due to the injection of 2D * $\hbox {TB}_{\mathrm{chelating}}$.

The speciation simulations (see Fig. 3) showed that when oxalic acid was added to half-diluted geothermal brine, 15% of this chelating agent remained uncomplexed as $\hbox {oxalate}^{{2-}}$. Consequently, this anion reacted with the metals present in the in situ geothermal fluid and formed different complexes. The interaction between 2D * $\hbox {TB}_{\mathrm{EDTA}}$ and TB is also partially endothermic and partially endothermic. However, the exothermic peaks are much smaller than the ones obtained with oxalic acid. This could indicate that EDTA in 2D * $\hbox {TB}_{\mathrm{EDTA}}$ complexed to a lower extent with the in situ geothermal fluid metals. The results from the speciation simulations (see Fig. 3) showed that EDTA complexed to a great extent with iron, forming $\hbox {[Fe(EDTA)]}^{{2-}}$ and calcium, forming $\hbox {[Ca(EDTA)]}^{{2-}}$. Consequently, a smaller amount of EDTA could complex with the metals in TB. The thermogram for 2D * $\hbox {TB}_{\mathrm{EDDS}}$ into TB indicates that the interaction is initially endothermic probably due to the dilution of the in situ brine. Then, it becomes mainly exothermic as it was observed for the previous complexing agents. EDDS shows a higher complexation degree with the in situ geothermal brine than EDTA. This could be because EDTA was already highly complexed in 2D * TB. Thus, it is logical that EDTA reacted in a lesser extent with TB. The last tested chelating agent for iron control was humic acids (HAs) that resulted from microbial and chemical transformations of organic debris. HAs are nonvolatile poly-electric acids with molecular weights ranging from 500 to 5000 g (Stumm and Morgan 1996). The presence of carboxylic, phenolic, quinone, and amino functional groups give HAs ionic exchange/oxidation–reduction characteristics (Zhang and Zhou 2019). Figure 8 shows the heat response obtained for 2D * $\hbox {TB}_{\mathrm{Humic}}$ into TB. Similarly to the previous agents, uncomplexed humic acids form complexes with the metals in the in situ brine. It is displayed in Fig. 3 that 72% of the humic acids remained as a free ligand and therefore it could interact with the in situ geothermal brine.

The endothermic peaks displayed in Fig. 8 were associated with a disturbance in the hydrogen bonding (HB). As observed, the heights of the peaks in the thermograms presented in Fig. 8 are smaller than the ones shown in Fig. 5. The lower heat flow for the fluid–fluid interactions in comparison to the rock–fluid interactions could indicate that the chelating agents are also interacting with the rock particles. A similar trend was observed in our previous work (Cobos and Søgaard 2021), in which citric acid was used as a complexing agent. Fredd and Fogler (1998) mentioned that the dissolution of calcite in presence of chelating agents such as EDTA depends on the kinetics of the chelation reactions and pH. Those authors introduced a surface chelation mechanism to describe the dissolution kinetics of calcite. The proposed mechanism indicates that the chelating agent adsorbs onto the active sites of the mineral lattice. This in turn weakens the bonds by forming complexes, which are subsequently transported away from the mineral lattice to the bulk solution. Consequently, the mineral dissolution is a combined chelating effect and $\hbox {H}^{+}$ attack driven by entropy due to the increment of the dissolved aqueous species in the bulk solution.

**Fig. 9:**
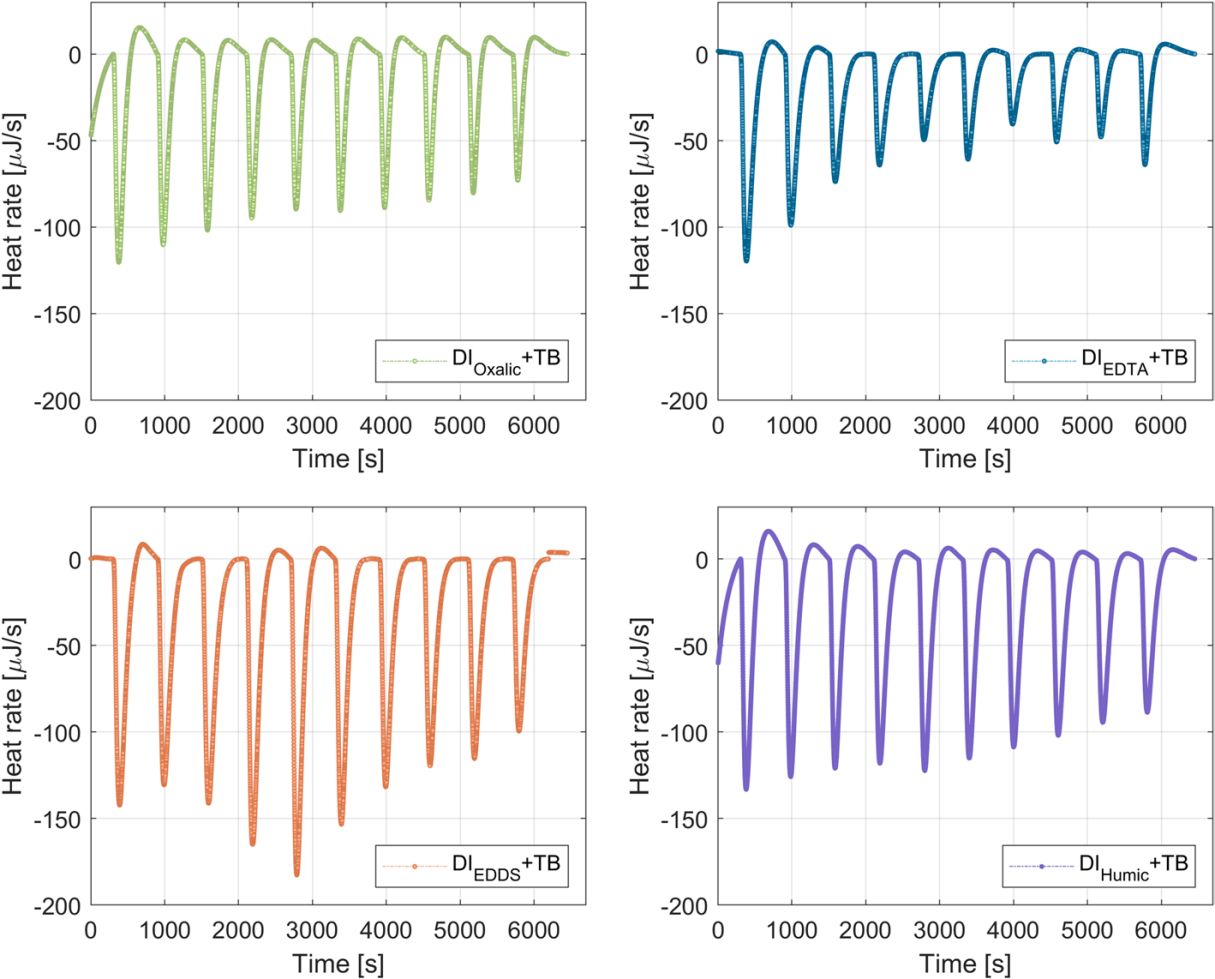
Thermograms for the interaction of $\hbox {DI}_{\mathrm{chelating}}$ and TB

Figure 9 displays the thermograms obtained for the injection of $\hbox {DI}_{\mathrm{chelating}}$ into TB. As observed the interaction of $\hbox {DI}_{\mathrm{chelating}}$ with TB is also endothermic as for the Berea + TB + $\hbox {DI}_{\mathrm{chelating}}$ system. This could indicate that the dilution of the in situ brine is the main process that occurs when $\hbox {DI}_{\mathrm{chelating}}$ contacts the reservoir.

**Fig. 10:**
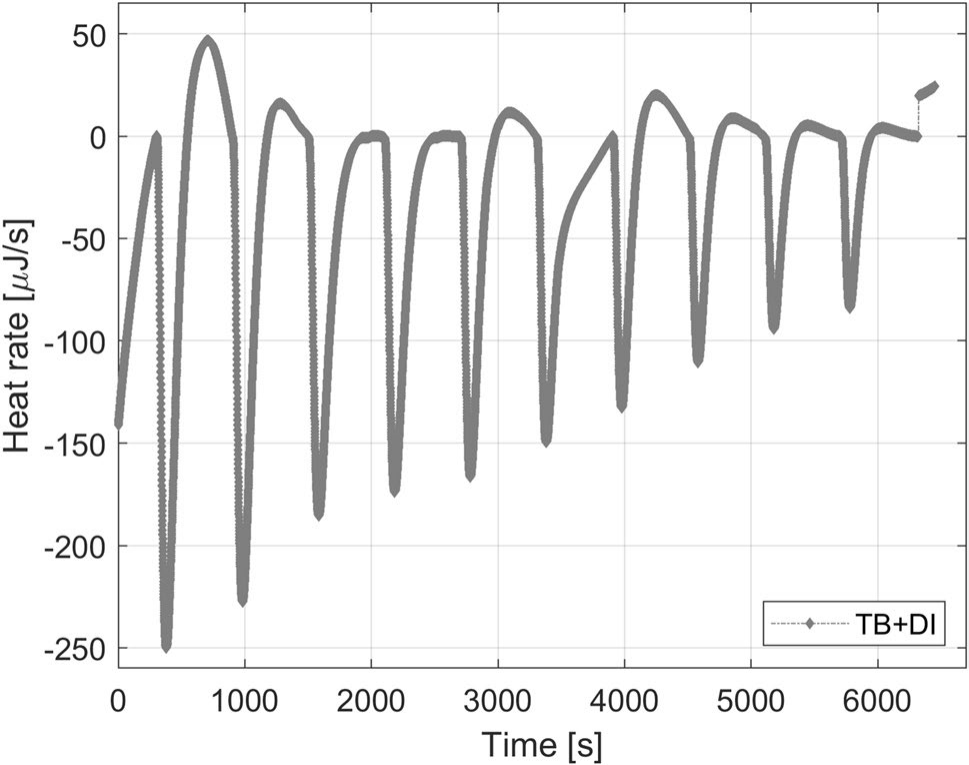
Thermograms for the interaction of DI and TB

It was observed previously that the dilution of the geothermal brine (endothermic process) masked the formation of complexes. In order to test that hypothesis, deionized water was injected into TB. Figure 10 shows that the interaction between DI water and TB is effectively endothermic. Notice that the peaks for this interaction are larger than for $\hbox {DI}_{\mathrm{chelating}}$ + TB. Consequently, the addition of a diluted fluid with chelating agents into a highly concentrated brine masked the exothermic formation of complexes.

The integration of the heat rate observed in Figs. 8 and 9 over the baseline (time) gives the heat developed by each injection of 9.947 μl of 2D * $\hbox {TB}_{\mathrm{chelating}}$ or $\hbox {DI}_{\mathrm{chelating}}$ into TB. Table 6 summarizes the results from the integration of the area between peaks and the baseline.

**Table 6:** Heat values for fluid–fluid (2D * $\hbox {TB}_{\mathrm{chelating}}$ or $\hbox {DI}_{\mathrm{chelating}}$ into TB)

Inj	Oxalic into TB (mJ)		EDTA into TB (mJ)		EDDS into TB (mJ)		Humic into TB (mJ)	
	2D * $\hbox {TB}_{\mathrm{oxalic}}$	$\hbox {DI}_{\mathrm{oxalic}}$	2D * $\hbox {TB}_{\mathrm{EDTA}}$	$\hbox {DI}_{\mathrm{EDTA}}$	2D * $\hbox {TB}_{\mathrm{EDDS}}$	$\hbox {DI}_{\mathrm{EDDS}}$	2D * $\hbox {TB}_{\mathrm{Humic}}$	$\hbox {DI}_{\mathrm{Humic}}$
1	2.15	10.40	3.95	15.75	0.44	19.18	0.89	13.13
2	1.01	11.22	5.43	13.79	− 0.34	19.86	0.01	14.64
3	0.31	9.93	4.18	11.28	− 1.26	22.54	− 0.96	13.92
4	− 0.18	8.64	3.42	9.60	− 1.83	22.33	− 1.55	15.80
5	− 0.52	7.70	2.62	7.39	− 1.96	23.25	− 1.71	15.46
6	− 0.37	7.27	2.06	7.68	− 1.96	21.46	− 1.43	14.81
7	− 0.64	6.58	1.97	5.66	− 1.83	18.51	− 0.83	14.14
8	− 0.82	5.85	1.78	5.57	− 1.90	17.05	− 0.13	13.67
9	− 0.45	5.44	1.65	5.12	− 1.82	16.38	− 0.19	13.12
10	− 0.07	4.45	1.96	5.02	− 1.22	14.66	− 0.69	11.38

The results presented in Table 6 clearly show that the heat response for 2D * $\hbox {TB}_{\mathrm{EDTA}}$ into TB is mainly endothermic and driven by the dilution of the in situ geothermal brine. In other words, EDTA already complexed with the metals in 2D * TB. Consequently, this chelating agent could keep more iron in solution due to its ability of forming highly stable water-soluble metal complexes. Beiyuan et al. (2018) reported that EDTA shows a high lead (Pb) extraction efficiency. This could be of a great benefit for geothermal plants in which galvanic corrosion due to dissolved $\hbox {Pb}^{{2+}}$ (e.g., Margretheholm geothermal plant in Denmark) (Guddat and Juul 2019) and PbS scaling problems (Neustadt-Glewe in northern Germany) (Heberling et al. 2017) have been reported. The environmental risk associated with the usage of EDTA due to its low non-biodegradable nature is negligible because EDTA should be added to the reinjection fluid that is not in contact with fragile ecosystems. EDDS is a potential alternative to EDTA because it shows a high performance in terms of rock–fluid and fluid–fluid interactions. Prete et al. (2021) mentioned that EDDS can replace EDTA when used for metal extraction due to its biodegradation capacity. According to Chauhan et al. (2012), EDDS not only have a high biodegradability but also a good recovery. These authors found that more than 96% EDDS could be recovered from nickel extraction. The main limitation with the usage of EDDS is the high cost (5000 GBP per ton) (Nowack et al. 2003).

## Conclusions

- Chemical-equilibrium diagrams indicate that hexadentate EDTA is more prone to form complexes with iron than bidentate oxalate ligand.
- Isothermal titration calorimetry (ITC) experiments display that the total heat for the formation of soluble metal–ligand complexes in the rock–brine system follows: EDTA > EDDS > oxalic acid > humic acid.
- EDTA is the best complexing agent for the reinjection of diluted geothermal brine into the reservoir since it forms more complexes in 2D * TB than the other chelating agents. Moreover, it could be used to avoid scaling problems in the reinjection wells.
- EDDS is an alternative complexing agent for the reinjection of diluted geothermal brine. This chelating agent has a high biodegradability and also a good recovery when used for metal extraction.
- The total interaction for diluted Thisted brine with humic acid (2D * $\hbox {TB}_{\mathrm{humic}}$) is highly entropic (highest endothermic enthalpy) due to conformational changes.

## Data Availability

The raw microcalorimetry data are available.
